# Single-Cell Characterization of Hepatic CD8^+^ T Cells in a Murine Model of Primary Biliary Cholangitis

**DOI:** 10.3389/fimmu.2022.860311

**Published:** 2022-04-20

**Authors:** Yichen Han, Zhen-Hua Bian, Si-Yu Yang, Cheng-Bo Wang, Liang Li, Yan-Qing Yang, Aftab A. Ansari, M. Eric Gershwin, Xiaofeng Zeng, Zhe-Xiong Lian, Zhi-Bin Zhao

**Affiliations:** ^1^Department of Rheumatology, Peking Union Medical College Hospital, Peking Union Medical College and Chinese Academy of Medical Sciences, National Clinical Research Center for Dermatologic and Immunologic Diseases, Ministry of Science and Technology, Key Laboratory of Rheumatology and Clinical Immunology, Ministry of Education, Beijing, China; ^2^School of Medicine, South China University of Technology, Guangzhou, China; ^3^Medical Research Center, Guangdong Provincial People’s Hospital, Guangdong Academy of Medical Sciences, Guangzhou, China; ^4^Department of Oncology of the First Affiliated Hospital, Chinese Academy of Sciences Key Laboratory of Innate Immunity and Chronic Disease, Division of Life Sciences and Medicine, University of Science and Technology of China, Hefei, China; ^5^Division of Rheumatology, Allergy and Clinical Immunology, University of California at Davis School of Medicine, Davis, CA, United States

**Keywords:** primary biliary cholangitis, CD8^+^ T cells, single-cell sequencing, TCR repertoire, liver

## Abstract

Primary biliary cholangitis (PBC), an organ-specific autoimmune disease, is characterized by injury to small bile ducts, inflammatory cell infiltrates within the liver, progressive cholestasis, and in some cases, cirrhosis with unclear pathogenesis. We aimed to clarify the importance role of hepatic immunce cells in the pathogenesis of human and experimental PBC.The dominant-negative TGFβ receptor type II transgenic (dnTGFβRII) mice, a well-studied and established murine model of PBC were used to identify changes of immune cells, especially the pathogenic CD8^+^ T cells. The high-throughput single-cell RNA sequencing technology were applied and found functional heterogeneity among the hepatic CD8^+^ T cells subsets in dnTGFβRII mice. CD8^+^ T cells were confirmed the key cells leading to the pathogenesis of PBC in dnTGFβRII mice, and identified the terminally differentiated CD8αα T cells and CD8αβ T cell subsets in the liver of dnTGFβRII mice. While terminally differentiated CD8αα T cells have higher cytokine production ability and cytotoxicity, the terminally differentiated CD8αβ T cells retain their proliferative profile. Our work suggests that there are developmental and differentiated trajectories of pathogenic CD8^+^ T cell subsets in the pathogenesis of PBC. A further clarification of their roles would be helpful to our understanding of the pathogenesis of PBC and may potentially lead to identifying novel therapeutic modalities.

## Introduction

Primary biliary cholangitis (PBC) is a chronic, destructive, autoimmune and cholestatic liver disease that predominantly affects middle-aged women. Results of studies so far suggest that there is a breakdown of immune tolerance in the liver of PBC patients that leads to the targeting of small-medium bile ducts which is accompanied by portal inflammation and progressive cholestasis (1, 2). The major symptoms of PBC are fatigue and itching. Development of PBC is hypothesized to be due to a combination of genetic risk factors and environmental triggers, also cellular, immunological and physiological response to biliary injury (3). Epidemiology results showed that PBC is strongly associated with recurrent urinary tract infection and 48% of PBC patients had prior recurrent urinary tract infections vs 31% of controls (4). Although, ursodesoxycholic acid (UDCA) has been approved by FDA as a first-line treatment for PBC patients, approximately 40% of patients fail to respond to UDCA treatment, leading to liver fibrosis and cirrhosis that ultimately results in liver transplantation as being the only therapeutic option (5). The lack of response to UDCA is mainly attributed to our lack of an understanding of the mechanism of the pathogenesis of this disease (6). Previous studies indicate that PBC is most likely a result of a combination of genetic susceptibility and environmental factors (7). Histological and phenotypic studies have reported that there are significant changes in the lineages of T cells (8), B cells (9), DCs (10), NK cells (11, 12) and monocytes/macrophages (13, 14), both in the liver and peripheral blood of patients with PBC. One of the highlights of these studies has been the identification of PDC-E2-specific autoreactive cytotoxic T lymphocytes uniquely present in liver tissues of PBC patients (8). However, the detailed immunological mechanism of PBC remains elusive.

The dnTGFβRII (TG) mice is a well-established murine model of human PBC with transgenic expression of a dominant negative form of human TGF-β receptor type II under the control of the mouse CD4 promoter, which leads to the blockade of TGFβ signaling pathway in CD4^+^ T and CD8^+^ T cells (15). The TG mice have been used as an autoimmune cholangitis murine model for exploring the pathogenesis of the disease including the natural history of PBC (16). Utilizing this murine model of human PBC, we have previously documented a major role for CD8^+^ T cells in the pathogensis of PBC by performing adoptive transfer experiments. Thus, the adoptive transfer of CD8^+^ T cells from TG to normal mice led to signficant damage to the small-medium size bile ducts, a hallmark of human PBC (17). These findings led to a series of subsequent studies that were focused on the fine characterization of these CD8^+^ T cells (18–20). Since all the data that we have so far obtained using the TG model and studies of tissues from PBC patients suggest a vital role of CD8^+^ T cells in the pathogenesis of PBC, in the present study, we used single-cell RNA sequencing (scRNA-seq) of liver CD8^+^ T cells to move this field forward. Results of the studies reported herein show that there is a subpopulation CD8αα T cells that may play a unique role in the pathogenesis of PBC. We submit that these findings may be helpful in the discovery of novel therapeutic interventions in the future.

## Materials and Methods

### Mice

C57BL/6 mice (B6) were purchased from Hunan SJA Laboratory Animal Co., LTD (Hunan, China). TG mice (B6. Cg-Tg (Cd4-TGFBR2)16Flv/J, TG) were initially purchased from the Jackson Laboratory (Bar Harbor, Maine, USA) and housed at the University of California at Davis and then transported to South China University of Technology, Guangzhou, China. CD1d^-/-^ mice were kindly provided by Prof. Li Bai (University of Science and Technology of China). All mice were housed in individually ventilated cages under specific pathogen-free conditions in a controlled environment (20°C-26°C, 40%-70% humidity, and a 12-h day/night cycle). All animal experimental protocols were performed according to the Guide for the Care and Use of Laboratory Animals and approved by the Ethics Committee on Animal Use at South China University of Technology.

### Hepatic Mononuclear Cells Preparation

8 to 9 week old TG and B6 mice were sacrificed, and hepatic mononuclear cells (HMNCs) were prepared as previously described (21) with minor adjustment. Briefly, liver tissues were homogenized in phosphate-buffered saline (PBS) containing 0.2% bovine serum albumin (BSA) and passed through a steel mesh. Liver mononuclear cells were isolated from hepatocytes through centrifugation with 40% Percoll (GE Healthcare, Little Chalfont, UK) at room temperature, followed by removal of red blood cells using Red Blood Cell Lysis Buffer (Beyotime, Shanghai, China). Cells were counted and confirmed for viability using trypan blue.

### Flow Cytometry and Cell Sorting

For cell surface marker staining, single-cell suspensions were incubated with anti-mouse CD16/32 (BioLegend, San Diego, CA, USA) at 4°C for 15 min for FcR blocking, and then incubated with a variety of fluorochrome-conjugated antibodies at 4°C for 20 min. The fluorochrome conjugated antibodies utilized included anti-CD44 FITC (IM7), anti-CD62L PerCP-Cy5.5 (MEL-14), anti-NK1.1 PE-Cyanine7 (PK136), anti-CD3 APC-Cyanine7 (17A2), anti-KLRG1 APC (2F1/KLRG-1), anti-CD3 Brilliant Violet 421™ (17A2), anti-CD8β Alexa Fluor^®^ 700 (YTS156.7.7), anti-NK1.1 PE-Cyanine5 (PK136), anti-CD8α Brilliant Violet 785™ (53-6.7), anti-CD44 Alexa Fluor^®^ 647 (IM7), anti-CD44 Brilliant Violet 711™ (IM7), anti-TIGIT APC (1G9), anti-CD38 APC-Cyanine7 (90), anti-CD73 PE-Cyanine7 (TY/11.8), anti-CD69 PE-Dazzle™ 594 (H1.2F3), anti-CD4 PE (GK1.5), anti-CD8α PE-Cyanine7 (53-6.7), anti-NK1.1 APC (PK136) and anti-CD8α FITC (53-6.7) that were all purchased from BioLegend, Inc (San Diego, CA). Anti-CD4 BUV563 (GK1.5), anti-CD45.2 V500 (104), anti-CD4 Brilliant Violet 510™ (RM4-5) and anti-CD62L BUV737 were purchased from BD Biosciences (San Jose, CA, USA). The Brilliant Violet 421™ and PE (PBS-57 loaded) conjugated anti-CD1d-tetramer reagents were kindly provided by the U.S. NIH Tetramer Core Facility, Bethesda, MD). After incubating, washing, and resuspending, the cells were analyzed using the BD LSRFortessa or BD Verse and the purified cell populations were isolated using the FACS Aria SORP cell sorter system (BD Biosciences). Original data were analyzed by FlowJo software (V10.7.2), Tree Star, Ashland OR.

### Adoptive Transfer

Splenic and hepatic CD8^+^ T cells were isolated from 8-9 week old TG mice and transferred into Rag1^-/-^ mice as described (17). Briefly, splenic, and hepatic CD8^+^ T cells were purified from mononuclear cells respectively by positive selection whereby the cells were first incubated with FITC-CD8α followed by incubation with anti-FITC microbeads(Miltenyi Biotec, Bergisch Gladbach, Germany). Splenic naïve CD8^+^ T cells, central memory CD8^+^ T cells and effector memory CD8^+^ T cells were isolated from TG mice using standard cell sorter techniques. 1×10^6^ cells of each population were transferred into Rag1^-/-^ mice by intravenous injection.

### Multi-Color Immunohistochemistry

The livers from 8-9 weed old TG and B6 mice were fixed with 4% paraformaldehyde and embedded in paraffin. These liver paraffin blocks were cut into 4 μm sections and adhered onto glass slides. The paraffin sections were placed in a 60°C oven for 2 hours before deparaffinization in xylene followed by successive sequential rehydration in 100%, 95%, 80%, 70% alcohol. Antigen was retrieved by 0.01 mol/L citric acid sodium citrate buffer (pH 6.0) at high temperature using a research microwave oven for 7-8 min followed by incubation at low temperature for 15 min. Endogenous peroxidase was inactivated by incubation in 3% H_2_O_2_-methanol for 10 min. After blocking nonspecific staining using 10% goat serum for 20 min, the sections were incubated with primary antibodies in a humidified chamber at 4°C overnight, avoiding light. Following incubation with horseradish peroxidase (HRP) and tyramide signal amplification (TSA) the next day, antigen retrieval was performed by incubation with citric acid buffer and repeating the above process. The primary panel antibodies and IHC metrics used were: 520nm-CD8α (1: 100, 70306, CST), 620nm-CD8β (1:100, ab228965, Abcam), 690nm-CK19 (1:3000, ab52625, Abcam), and DAPI (Beyotime). Slides were imaged with Vectra^®^ Polaris™ Automated Quantitative Pathology Imaging System (Akoya Biosciences) and quantified by inform 2.4.8 software (Akoya Biosciences).

### RNA Isolation and Sequencing

CD8αα and CD8αβ T cells were sorted using FACS Aria SORP sorting system, lysed and stored in Trizol reagent (Invitrogen, USA) at -80°C for RNA isolation and subsequent RNA sequence. For bulk RNA sequencing, RNA was extracted from these two lineages of cells according to the RNeasy MinElute Cleanup Kit (74204, QIAGEN)’s protocol. RNA-sequencing data sets were deposited into the Gene Expression Omnibus of the NCBI under accession number GSE186399.

### Oligo Conjugated Anti-Mouse Antibodies

Aliquots of 2 × 10^5^ hepatic mononuclear cells were stained with 1 µL of a combination of the following oligo conjugated anti-mouse antibodies (AMM2032-CD103, AMM2008-CD11c, AMM2069-CD122, AMM2095-CD137, AMM2033-CD138, AMM2003-CD16/32, AMM2097-CD172a, AMM2047-CD184, AMM2061-CD197, AMM2104-CD200, AMM2048-CD223, AMM2057-CD23, AMM2040-CD24, AMM2024-CD279, AMM2016-CD28, AMM2036-CD335, AMM2084-CD357, AMM2103-CD366, AMM2051-CD41, AMM2041-CD43, AMM2010-CD44, AMM2014-CD45.2, AMM2006-CD45R, AMM2050-CD49a, AMM2034-CD49b, AMM2075-CD49d, AMM2043-CD5, AMM2018-CD62L, AMM2022-CD69, AMM2059-CD71, AMM2044-CD90.1, AMM2035-CD90.2, AMM2030-CD95, AMM2096-CD9, AMM2009-Ly6G, AMM2021-TCRβ, AMM2028-TER, AMM2087-TIGIT, AMM2007-CD19, AMM2067-CD1d, AMM2012-CD25, AMM2001-CD3, AMM2002-CD4, AMM2049-IgD, AMM2031-IgM, AMM2017-NK1.1, AMM2066-TCRVα2, AMM2092-TCRVβ5.1-5.2, and AMM2077-CD8β).

### Single Cell Capture, cDNA Synthesis and Library Preparation

Single-cell capture was achieved by Poisson distribution of a single-cell suspension across >200,000 microwells (BD Rhapsody™). Beads with oligonucleotide barcodes were added to saturation to pair with the beads with the cells in the microwells. Cell-lysis buffer was added to lyse the cell to allow barcode conjugated beads to capture poly-adenylated molecules, including mRNA, AbSeq targets and sample tag. Reverse transcription was performed on beads for WTA and AbSeq and Sample Tag Library Preparation. Finally, the library was sequenced using Novaseq platform (Illumina, San Diego, CA) on a 150 bp paired-end run.

### Singe-Cell RNA Sequencing Data Analysis

The raw sequencing data of gene expression and extracellular protein expression were aligned with the mm10 mouse reference genome, using STAR algorithm in BD Rhapsody analysis pipeline (version 1.9.2β, BD). A data matrix with 13,577 features (including 13,528 genes and 49 proteins) and 11,929 cells was obtained after filtering. The data matrix was transformed as a seurat object with Seurat package (version 4.0.1). The batch between the datasets was removed with CCA in Seurat package. Clustering was used to identify subsets within immune cells. Pseudotime analysis was performed using the monocle R package (version 2.18.0) with normalized data to deduce the relationship among cell subsets. Raw sequencing datasets were deposited into the Gene Expression Omnibus of the NCBI under accession number GSE186333.

### TCR Repertoire Analysis

To analyze the TCR repertoire from the RNA sequencing data, the TCR repertoire dataset was determined by MixCR (version 3.0.12) and imported into R language (Version 4.0.1) to calculate percentage and TCR repertoire diversity.

### Statistical Analysis

All data are presented as mean ± SD, except for special instructions. Statistical analysis was performed by a two-tailed unpaired Student’s t test in GraphPad Prism. P < 0.05 was considered statistically significant differences (*, P < 0.05; **, P < 0.01; and ***, P < 0.001).

## Results

### CD8^+^ T Cells Are the Key Cells Leading to the Pathogenesis of PBC in the TG Mouse Model

**Figure 1:**
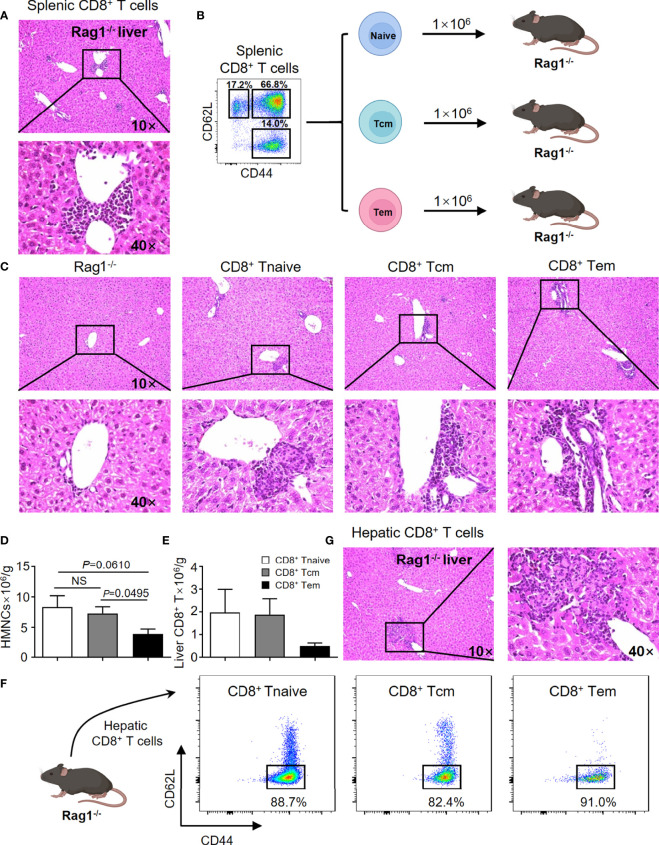
CD8^+^ T cells are the key cells leading to the pathogenesis of PBC in the TG murine model. **(A)** Representative H&E staining of liver sections of Rag1^-/-^ mice that were recipients passively transferred splenic CD8^+^ T cells from TG mice. **(B)** Schematic representation of the adoptively transferred splenic CD8^+^ T subpopulations into Rag1^-/-^ mice. **(C)** Representative H&E staining of liver sections of Rag1^-/-^ mice that were recipients of passively transferred splenic CD8^+^ T subpopulations from TG mice. **(D)** Number of hepatic mononuclear cells of recipients, data are presented as mean ± SEM. **(E)** Number of hepatic CD8^+^ T cells of recipients, data are presented as mean ± SEM. **(F)** Representative components of hepatic CD8^+^ T cells of Rag1^-/-^ mice that were recipients of passively transferred splenic CD8^+^ T cells from TG mice. **(G)** Representative H&E staining of liver sections of Rag1^-/-^ mice that were recipients of passively transferred hepatic CD8^+^ T cells from TG mice. NS, No significance.

In efforts to confirm previous findings and to provide baseline data for the current study, the adoptive transfer studies were repeated. Thus, adoptive transfer of splenic CD8^+^ T cells from TG mice induced autoimmune cholangitis in Rag1^-/-^ mice (**Figure 1A**). In efforts to narrow down the subset of CD8^+^ T cells involved, we isolated naïve CD8^+^ T cells (Tnaive, CD44^-^CD62L^+^), central memory CD8^+^ T cells (Tcm, CD44^+^CD62L^+^) and effector memory CD8^+^ T cells (Tem, CD44^+^CD62L^-^), and transferred them to Rag1^-/-^ mice respectively (**Figure 1B**). Of interest was the finding that while all three CD8^+^ T cell subsets induced autoimmune cholangitis, liver inflammation and liver infiltration of CD8^+^ T cells was the mildest in the group that were recipients of splenic CD8^+^ Tem cells (**Figures 1C–F**). To further confirm the pathogenic role of CD8^+^ T cells in PBC, we demonstrated that adoptive transfer of hepatic CD8^+^ T cells from TG mice also induced autoimmune cholangitis in Rag1^-/-^ mice (**Figure 1G**).

### Single-Cell Multi-Omics Analysis of Hepatic Immune Cells in TG and WT Mice

**Figure 2:**
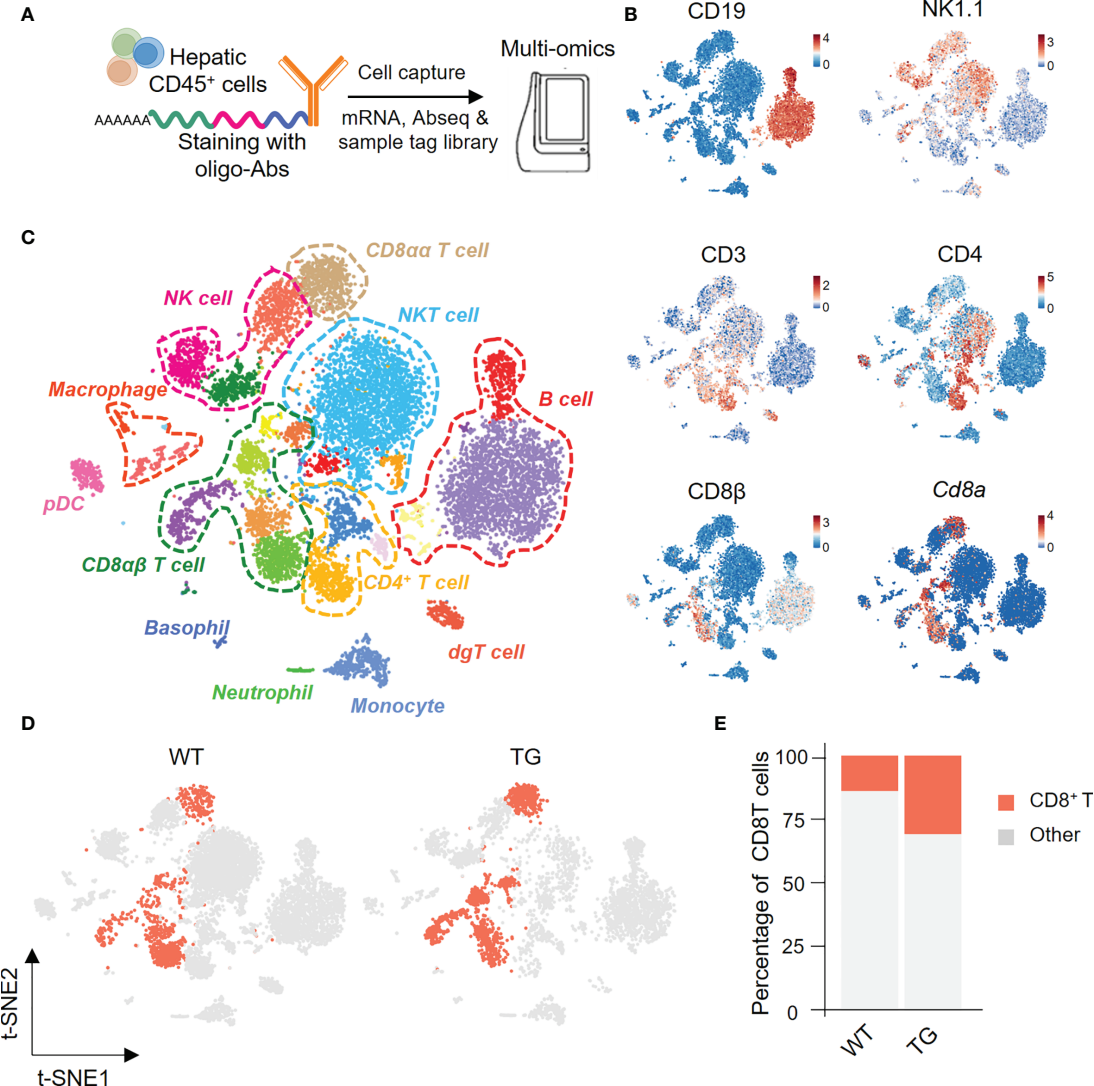
Single-cell multi-omics analysis of hepatic immune cells from TG and control mice. **(A)** Schematic representation of the workflow for single cell preparation, labels for antibodies, cell capture and library preparation. **(B)** The immune cell subsets identified with a combination of antibodies and genes, include CD19, NK1.1, CD3, CD4, CD8β and Cd8α. **(C)** Merged t-SNE plots of twelve immune subsets in livers of WT and TG mice. **(D)** CD8^+^ T cells from WT and TG mice highlighted with Split t-SNE plots. **(E)** Percentage of CD8^+^ T cells in WT and TG mice.

We next isolated highly enriched subpopulations of immune cell populations from the livers from TG and WT mice and performed single-cell RNA sequencing that included the identification of surface proteins uniquely expressed by each cell subset and mRNA expression simultaneously. In summary, we obtained a total 11,929 CD45^+^ hepatic immune cells, and detected the expression levels of 13,528 genes and 49 proteins (**Figure 2A**). Based on the surface protein and mRNA expression profiles (**Figure 2B**), we divided CD45^+^ cells into 12 cell subtypes. The subtypes included CD8αα T cells (CD3, *Cd8a*), CD8αβ T cells (CD3, CD8β, *Cd8a*), CD4 T cells (CD3, CD4), NKT cells (CD3, NK1.1), NK cells (NK1.1), and B cells (CD19) (**Figure 2C**). We then focused on CD8^+^ T cells and split the merged t-SNE plot by sample into WT and TG mice (**Figure 2D**). The percentage of hepatic CD8^+^ T cells was higher in TG mice than the WT mice (**Figure 2E**).

### Differential Gene Expression Profile of Hepatic Effector Memory CD8^+^ T Cells

**Figure 3:**
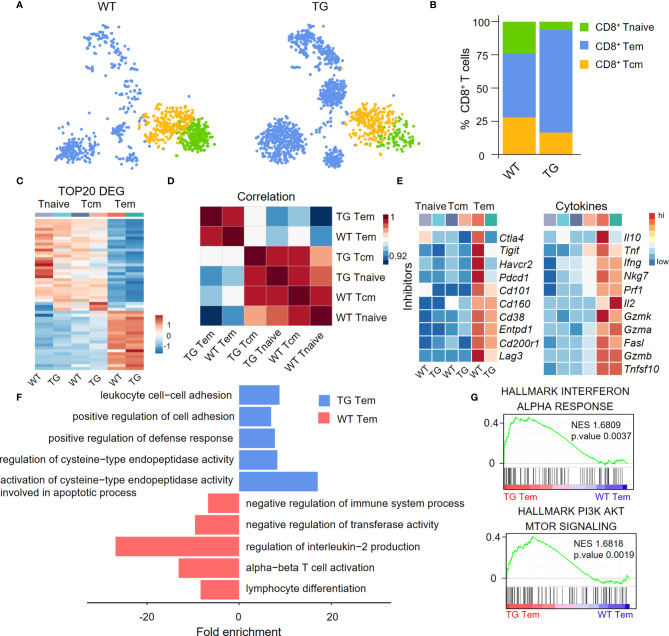
Characterization of liver CD8^+^ Tem cells in TG mice. **(A)** CD8^+^ T cell subpopulations from WT and TG mice, including naïve CD8**^+^** T cells, central memory CD8**^+^** T cells and effector memory CD8**^+^** T cells, shown with split t-SNE plots. **(B)** Percentage of CD8^+^ T subpopulations of WT and TG mice. **(C)** Scaled average expression of the top 20 upregulated differentially expressed genes for each CD8^+^ T subpopulations. Individual cells are represented on the horizontal axis and grouped by cluster. **(D)** The correlation analysis of average gene expression among CD8^+^ T subpopulation of WT and TG mice. **(E)** Scaled average expression of inhibitory checkpoint molecules and cytokines by CD8^+^ T subpopulations from WT and TG mice. **(F)** GO analysis of the upregulated genes in effector memory CD8**^+^** T cells of WT and TG mice. **(G)** GSEA analysis of Interferon-α response and PI3K-AKT-mTOR signaling for effector memory CD8**^+^** T cells between TG and WT mice. The normalized enrichment score (NES), original p value and FDR adjusted p values (q.value) are displayed.

We divided CD8^+^ T cells into CD8**^+^** Tnaive cells, central memory CD8**^+^** T cells (CD8^+^ Tcm cells) and effector memory CD8**^+^** T cells (CD8^+^ Tem cells) according to their functional gene expression profiles (**Figure 3A**). The percentage of CD8^+^ Tem cells was higher in the liver of TG mice than the WT mice (**Figure 3B**). The expression patterns of the top 20 upregulated cell type-specific genes and correlation analysis showed that CD8^+^ Tem cells have unique gene expression profiles (**Figures 3C, D**). In addition, CD8^+^ Tem cells from both WT and TG mice expressed higher relative levels of immune checkpoint molecules and pro-inflammatory cytokines than CD8^+^ Tnaive cells and CD8^+^ Tcm cells (**Figure 3E**). Moreover, several immune checkpoint molecules such as Ctla4, Tigit, Havcr2, and Pdcd1 were more downregulated in CD8^+^ Tem cells from TG mice than WT mice. Importantly, Gene Ontology (GO) analysis showed that CD8^+^ Tem cells from the liver of TG mice express a relatively higher level of cell activation-related genes, including genes involved in leukocyte cell-cell adhesion, positive regulation of cell adhesion and positive regulation of defense response (**Figure 3F**). GSEA analysis showed that the IFN-α response of CD8^+^ Tem cells was significantly higher in TG mice than WT mice, which suggests the presence of an auto-immune environment in the liver of TG mice. The PI3K-AKT-mTOR signaling pathway was highly enriched in CD8^+^ Tem cells of TG mice, indicating that these cells maintained a proliferating status. These observations, to some extent, explain the increase of these CD8^+^ Tem cells in the liver of TG mice (**Figure 3G**).

### Functional Heterogeneity of Effector Memory CD8^+^ T Cell Subsets in the Liver of TG Mice

**Figure 4:**
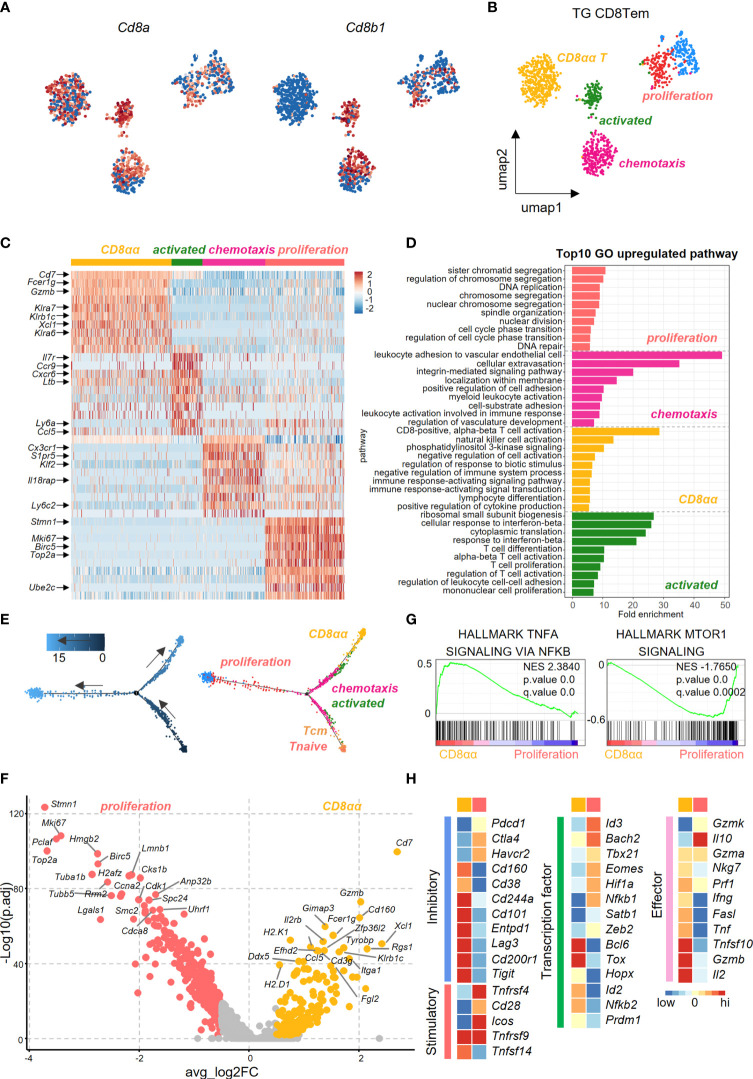
Transcriptional profiling of liver effector memory CD8^+^ T cells using scRNA-seq. **(A)** Normalized expression of *Cd8a* and *Cd8b1* in effector memory CD8**^+^** T cells of TG mice. **(B)** UMAP plot shows the five subpopulations of effector memory CD8**^+^** T cells in TG mice. **(C)** Scaled average expression of the top 10 upregulated differentially expressed genes for each effector memory CD8^+^ T subpopulations of TG mice. Individual cells are represented on the horizontal axis and grouped by cluster. **(D)** GO analysis of the upregulated genes in effector memory CD8^+^ T subpopulations of TG mice. **(E)** Pseudotime analysis showed the differentiation trajectory of CD8^+^ T subpopulations from TG mice. Cells on the tree are colored by pseudotime (left) and subpopulations (right). **(F)** Volcano plot displayed the DEGs between CD8αα and proliferation effector memory T cells with the labelling of key significant genes. Colorized entities represent passing the filter of p_val_adj < 0.01 and ave_log2FC > 0.5 (< -0.5) with golden (jacinth) gradient on ave_log2FC values. **(G)** GSEA analysis of TNFα-NFκB signaling and mTOR1 signaling between CD8αα and proliferation effector memory T cells. The normalized enrichment score (NES), original p value and FDR adjusted p values (q.value) are shown. **(H)** Scaled average expression of inhibitory and costimulatory checkpoint molecules (left), transcription factor molecules (middle) and effector molecules (right) for CD8^+^ T sub-population of CD8αα and proliferation effector memory T cells.

We further dissected CD8^+^ Tem cells into different subsets in efforts to identify the pathogenic clusters that maybe potentially involved in PBC. Uniform Manifold Approximation and Projection (UMAP) analysis identified five subpopulations within CD8^+^ Tem cells from the liver of TG mice. Among these five subpopulations, the highest percentage were the CD8αα T cells (**Figures 4A, B**). Heatmap revealed differences in the gene expression profile among subsets of CD8^+^ Tem cells (**Figure 4C**). GO analysis showed that the up-regulated genes in CD8αα T cells and the CD8αβ T cells are enriched in functions associated with immune cell activation, lymphocyte differentiation and positive regulation of cytokine production (**Figure 4D**). Pseudotime analysis showed that hepatic CD8^+^ Tem cells display a distinct differentiation trajectory, that includes the terminally differentiated CD8αα T cells and terminally differentiated CD8αβ T cells (**Figure 4E**).

As seen in **Figure 4F**, the volcano plot showed that there were differentially expressed genes (DEGs) between CD8αα Tem and proliferating CD8αβ Tem cells. Effector function genes such as *Cd7, Gzmb, Klra6/7, Klrb1c* were upregulated in CD8αα T cells, which indicates that these cells have a cytotoxicity profile (**Figure 4F**). Moreover, while the TNFα-NFκB signaling pathways were enriched in CD8αα T cells, the mTOR1 signaling pathways were enriched in the proliferating CD8αβ Tem cells (**Figure 4G**). In addition, while the CD8αα T cells showed enrichment for inhibitory checkpoint molecules and cytokines, the proliferating CD8αβ Tem cells were more enriched for co-stimulatory checkpoints (**Figure 4H**).

### Flow Cytometric Profiles of Hepatic Terminally Differentiated CD8αα T Cellsin TG Mice

**Figure 5:**
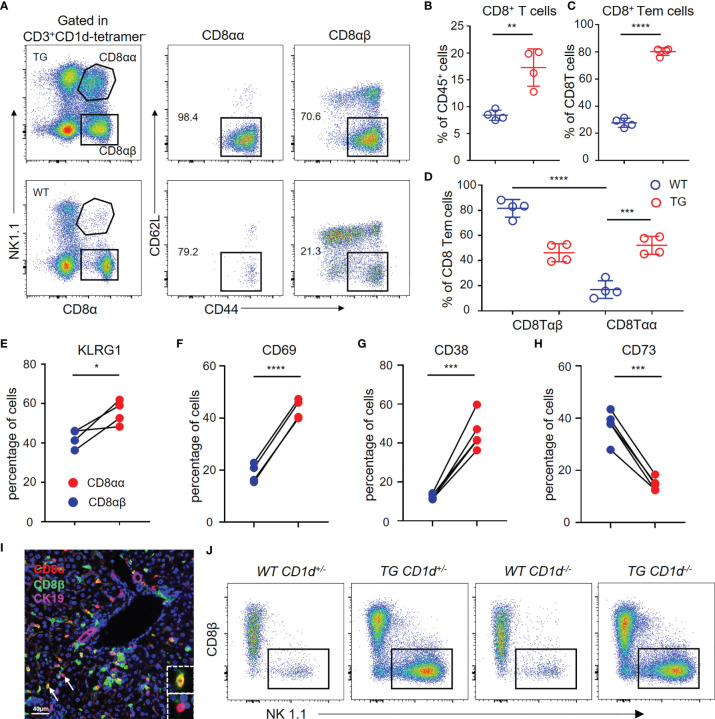
Hepatic CD8αα T cells in TG mice are terminally differentiated T cells. **(A)** Gating strategy used for flow cytometric examination of the percentage of CD8^+^ T cells sub-populations. **(B–D)** Percentage of CD8^+^ T cells, effector memory CD8**^+^** T cells and CD8αβ/CD8αα effector memory T cells in WT (n = 4) and TG mice (n = 4). **(E–H)** Frequency of CD8αβ and CD8αα T cells in TG mice that express KLRG1 (n = 4), CD69, CD38, and CD73 (n = 5). **(I)** Representative multiplex immunohistochemistry staining of CD8αα T cells in liver from TG mice for CD8α, CD8β and CK19 scale bar, 40 μm. **(J)** Representative FACS plot showing the percentage of NK1.1^+^ and CD8β^-^ CD8αα^+^ T cells within the CD3^+^CD1d-tetramer^-^ cells from the different groups of mice that included WT-CD1d^+/-^, TG-CD1d^+/-^, WT-CD1d^-/-^ and TG-CD1d^-/-^ mice. *, P<0.05; **, P<0.01; ***, P<0.001; ****, P<0.0001.

We used flow cytometry to analyze the hepatic CD8^+^ T cells from TG and WT mice (**Figure 5A**). The results demonstrated that CD8^+^ T cells and CD8^+^ Tem cells are significantly increased in the liver of TG mice (**Figures 5B, C**), which is consistent with the scRNA-seq data. Furthermore, the percentage of CD8αα T cells was much higher in the liver tissues from TG mice than WT mice (**Figure 5D**), and almost all CD8αα T cells were effector memory T cells. In addition, we detected the expression of molecules such as KLRG1, CD69 and CD38 related to CD8^+^ T cell activation that was highly expressed on CD8αα T cells of TG mice (**Figures 5E**–**G**), while the expression of CD73 was decreased (**Figure 5H**). We further confirmed the presence of CD8αα T cells in liver of dnTGFβII mice *via* multiplex immunohistochemistry (**Figure 5I**). Finally, we demonstrated that these cells are not NKT cells, and their development was independent of the CD1d molecule (**Figure 5J**).

### TCR Repertoire Diversity of CD8αα and CD8αβ T Cells in the Liver of TG Mice

**Figure 6:**
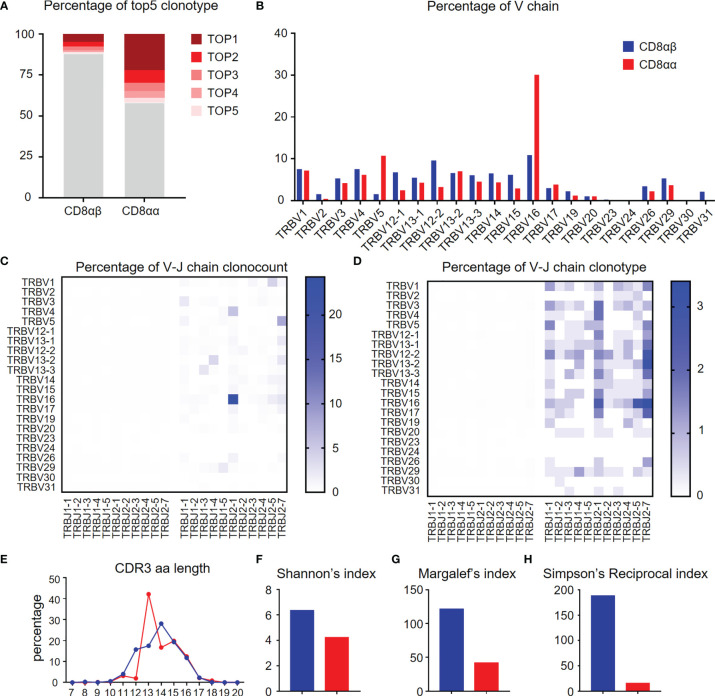
Diversity of TCR repertoire of hepatic CD8αα and CD8αβ T cells of TG mice. **(A)** Percentage of the top 5 TCR β-chain clonotypes in CD8αβ and CD8αα T cells from TG mice. **(B)** Frequency of functional V chains in CD8αβ and CD8αα T cells from TG mice. **(C, D)** Heat-maps of TCR β-chain V-J pairing usages in count (left) and type (right) for CD8αβ (left) and CD8αα (right) cells from TG mice. **(E)** CDR3 repertoire amino acid (AA) length distribution of TCRβ chain in CD8αβ and CD8αα cells from TG mice. **(F–H)** Diversity index of TCR β-chain repertoires in CD8αβ and CD8αα cells from TG mice, including Shannon’s index **(F)**, Margalef’s index **(G)** and Simpson’s Reciprocal index **(H)**.

TCR repertoire analysis provides data on the degree of clonal diversity of T cells and thus a measure of the potential ability of the T cells to respond to the number of antigens. The stimulation of antigens leads to the expansion of antigen-specific T cell clones. To compare the TCR repertoire diversity of hepatic CD8αα and CD8αβ T cells, we performed bulk RNA-seq to track the TCR clonotypes in TG mice. The data obtained shows that the top five TCRβ chain clonotypes were noted in CD8αβ and CD8αα T cells of TG mice, respectively. As seen, the CD8αα T cells exhibit more clonal expansion than CD8αβ T cells (**Figure 6A**). We also found that TRBV16 was a predominant clone in CD8αα T cells, which clearly contributes to the skewing profile of TCRs in this subset (**Figure 6B**). However, there was no predominant TRBV family in the clonal TCR β-chain of CD8αβ T cells. In addition, we noticed that TCR β-chain of CD8αα T cells was predominantly found on TRBV16-TRBJ2-1 in count and clonotype and mostly included TRBV16 and TRBJ2-7 (**Figures 6C, D**), which suggest the TCR β-chain V-J pairing is simple and repetitive in CD8αα T cells and is different from CD8αβ T cells. We found the predominant CDR3 length of TCR β-chain is shorter in CD8αα cells than CD8αβ cells (**Figure 6E**). We also performed three evaluation indices for the diversity of TCR β-chain repertoire, including Shannon’s index (**Figure 6F**), Margalef’s index (**Figure 6G**) and Simpson’s Reciprocal index (**Figure 6H**). All data obtained indicates that the TCRs of CD8αα T cells are skewed towards an oligoclonal profile. We interpret these data to indicate that perhaps autoantigen-specific CD8αβ T cells expand clonally, simultaneously down-regulate CD8β chain and differentiate into CD8αα T cells (22), possibly by a superantigen-driven mechanism.

## Discussion

PBC is a classic autoimmune liver disease, which is characterized by various clinical manifestations. To date, UDCA treatment remains the mainstream therapy for these patients. However, for reasons unclear, 20-40% of patients with PBC do not respond to such therapy. Although the outcome from clinical applications of traditional immunosuppressive therapies and other immune-based therapies such as rituximab, ustekinumab, NI-0801 and abatacept has been disappointing, we submit that the efficacy of novel immune modulation therapies needs further investigation (23). Previous studies mostly focused on low-throughput screening assays, in part limiting our understanding of the molecular mechanism behind the disease (18). It is reasoned that the availability of high-throughput screening assays, provides unique tools to advance our knowledge on the pathogenesis of this disease. In the present study, we utilized single-cell RNA sequencing technology to dissect the complex immune microenvironment in dnTGFβII mice, a well-recognized animal model of PBC, with the goal to refine our knowledge on the pathogenesis of autoimmune cholangitis.

In the present study, we used high-throughput single-cell RNA sequencing to show the heterogeneity of hepatic pathogenic CD8^+^ T cells in the murine model of human PBC. The results obtained indicate there may be a new differentiation pathway of CD8^+^ T cells within the liver. It is generally believed that CD8^+^ Tnaive cells become activated and differentiate into central memory CD8^+^ T cells following encounter with its cognate antigen, and then differentiate into effector memory CD8^+^ T cells (24). However, the pseudotime trajectories analysis of hepatic CD8^+^ T cells showed a divergence in effector memory cells. Not all effector memory CD8^+^ T cells proliferate. We submit that there is a small subset of CD8αβ T cells that downregulate CD8β and differentiate into CD8αα T cells, that have a TCR repertoire that seems oligoclonal and more simple and repetitive (**Figures S1A**–**C**).

CD8αα T cells were all effector memory (CD44^+^CD62L^-^) cells in TG mice. Surface antigen expression and transcriptome modules were assessed to characterize phenotype and functional features. Results of the studies reported herein show that CD8αα T cells to some extent play an unusual role in the pathogenesis of autoimmune cholangitis. Here, we isolated the top 10 genes that were remarkably upregulated or down-regulated in CD8αα T cells of TG mice. These genes to some extent provided us with a platform to speculate their effect in PBC pathogenesis and provide clues for future research. The highlights of the results include the findings that a) these CD8αα T cells are activated and may exert cytotoxic function during chronic disease progression b) These cells show a marked up-regulation of genes such as Gzmb, and IL2rb consistent with our view of a role for these cells in the pathogesis of this disease. It is well-known that high-doses of IL-2 can activate effector T cells to promote autoimmunity, which could result in the occurrence of autoimmune diseases, while low-doses of IL-2 can promote the development of a function that serves to control immune responses and maintain self-tolerance (25, 26). IL-2, also recognized as T cell growth factor, plays a pivotal role in the development, proliferation, survival, and differentiation of T cells. IL-2 is secreted from many immune cells such as T cells, activated dendritic cells (DCs), and NK cells, and performs its biological function *via* interacting with IL-2 receptors (IL-2Rs) (27, 28). In fact, IL-2R is a complex, consisting of three subunits: I IL-2Rα (CD25), IL-2Rβ (CD122) and IL-2Rγ (CD123). These subunits can, through different combinations, form three diverse receptors with different affinity for IL-2: low affinity receptor (IL-2α), intermediate affinity receptor (IL-2β and IL-2γ) and high affinity receptor (IL-2α, IL-2β and IL-2γ). CD8^+^ T cells and NK cells both express the intermediate affinity receptors, which mainly consists of two subunits, IL-2β and IL-2γ. Under the stimulation by cognate antigens, CD8^+^ T cells express the intermediate affinity receptors by high-dose IL-2 stimulation and exert their cytotoxic effects to promote the immune response. In addition, there are three key pathways that become activated upon the combination and interaction of IL-2 and IL-2R and include the JAK-STAT, PI3K/AKT/mTOR and MAPK/ERK pathways. These signaling pathways are very important factors for the differentiation, activation, survival, and proliferation of immune cells (25).

In addition, analysis of CDR3 length diversity and clonotype can be used to define the extent of clonal expansion within the TCR repertoire. The TCR β-chain CDR3 complex decreased in CD8αα T cells. Moreover, the top 5 clonotypes accounted for more than 40% of CD8αα T cells. Also, the V-J chain re-arrangement of CD8αα T cells were more simply and repetitive. These data collectively indicate that CD8αα T cells may be auto-responsive cells and could be activated and proliferate while encountering the autoantigen. In contrast to CD8αα T cells, the other subpopulation of CD8αβ T cells in terminal state of differentiation trajectory seems to be more focused on proliferation. The terminally differentiated CD8αβ T cells are enriched in glycolysis and phosphorylation, upregulated glycolysis related genes (*Eno1* (Enolase 1), *Hk2* (Hexokinases2), *Tpi1* (Triosephosphate Isomerase), *Ldha* (Lactate dehydrogenase A/B)) and ATP synthesis related genes (*Sdha/Sdhb* (Succinate dehydrogenase complex subunit A), *Ndufs7* (NADH: Ubiquinone Oxidoreductase Core Subunit S7), *Cox5a* (Cytochrome C Oxidase Subunit 5A)) to produce significant amounts of energy for proliferation. Furthermore, the terminally differentiated CD8αβ T cells showed an upregulation of lipid metabolism associated pathways that included the fatty acid metabolism and cholesterol homeostasis (**Figures S2A**–**C**), to provide intermediary metabolites for proliferation.

In addition, we had analyzed the relationship between hepatic CD8^+^ Trm (Tissue resident memory T cells) and CD8αα/CD8αβ and the results were included in the supplementary materials (i.e., **Figure S3**). In general, most of CD8αα Tem in TG mice had the phenotype of hepatic CD8^+^ Trm. However, only a small population of CD8αβ Tem resembled CD8^+^ Trm. We compared the upregulated genes (*Cxcr6*, *Itga1* and *Cxcr3*) and downregulated genes (*Klf2*, *Tcf7* and *S1pr1*) of hepatic CD8^+^ Trm in subpopulations of CD8^+^ Tem from TG mice (**Figure S3A**). The result suggests that the CD8αα Tem and activated CD8αβ Tem may resemble Trm. We also found the functional genes (like *Gzmb* and *Ifng*) and core transcription factors (*Notch1*, *Zfp683*(Hobit), *Prdm1*(Blimp-1), *Rbpj* and *Nr4a1*) of CD8^+^ Trm were mainly expressed in CD8αα Tem (**Figure S3B**). Moreover, we calculated the score of geneset (29) related to Trm phenotype and enriched in CD8αα Tem and activated CD8αβ Tem (**Figure S3C**). Based on the above results, we believed that hepatic CD8αα Tem cells in TG mice were tissue resident, and only activated Tem cells in CD8αβ Tem were similar to Trm. A recent staining study of clinicopathological tissues hinted that emperipolesis mediated by CD8^+^ T cells appears to be relevant to apoptosis of BEC, which may aggravate the further injury of interlobular bile ducts (30). In addition, a study reported that antigen-specific CD8^+^ T cells, which contact with hepatocytes, can control bile acid metabolism in a murine model of cholangitis, and that these effects partly depend on TNF and IFN-γ (31). As analyzed in our study, most hepatic CD8αα T cells in TG mice were effector memory like and had strong ability to produce IFN-γ (**Figures S4A, B**), suggesting that these cells may aggravate inflammation and/or reduce levels of unconjugated bile acids in the liver by IFN-γ at the same time. Moreover, a study found that patients with PBC also showed a markedly increased TIGIT^+^CD8^+^ T cells than the DCs (age- and sex- matched disease controls) and HCs (healthy controls) in peripheral blood. The expression of TIGIT was significantly increased in HLA-DR^+^CD8^+^ T cells compared with HLA-DR^-^CD8^+^ T cells (32), consistent with a previous research which found that the TIGIT upregulation might inhibit over-activated CD8^+^ T cells. We also had detected the high expression of TIGIT on CD8αα T cells in TG mice (**Figure S4C**), which may indicate these cells were over-activated by autoantigen to damage tissue and increase inflammation

In conclusion, we submit that our studies for the first time has led to the detection of a new differentiated trajectory of TCRαβ^+^ and CD8αα T cells in the liver of the murine model of human PBC by single-cell RNA sequencing. We found this subset of cells possesses an active and cytotoxic phenotype. We further discovered that they may have specificity for a restricted number of epitopes perhaps expressed by a similar group of antigens, which may introduce a new character participating in the PBC pathogenesis. In addition, we found a proliferation sub-population in CD8αβ effector memory T cells, which may explain the increased of CD8^+^ Tem in liver of TG mice. Further studies on the role of CD8^+^ T cells in autoimmune cholangitis will contribute our understanding of its pathogenesis.

## Data Availability Statement

The datasets presented in this study can be found in online repositories. The names of the repository/repositories and accession number(s) can be found below: https://www.ncbi.nlm.nih.gov/, GSE186333.

## Ethics Statement

The animal study was reviewed and approved by Guide for the Care and Use of Laboratory Animals, South China University of Technology or University of Science and Technology of China.

## Author Contributions

Z-XL, XZ, MG, and Z-BZ designed the experiments. YH and Z-HB carried out most of the experimental work, analyzed data and wrote the manuscript. S-YY, Y-QY and C-BW contributed to some experiments. LL, AA and MG edited and revised the manuscript. All authors contributed to the article and approved the submitted version.

## Funding

Financial support was provided by the National Key R&D Program of China (2017YFA0205600), Program for Guangdong Introducing Innovative and Enterpreneurial Teams (2017ZT07S054), the National Natural Science Foundation of China (82120108013, 81901652, 81801607), State Key Laboratory of Pathogenesis, Prevention, Treatment of Central Asian High Incidence Diseases Fund, China (SKL-HIDCA-2021-7), and CAMS Innovation Fund for Medical Sciences (2021-I2M-1-005).

## Conflict of Interest

The authors declare that the research was conducted in the absence of any commercial or financial relationships that could be construed as a potential conflict of interest.

## Publisher’s Note

All claims expressed in this article are solely those of the authors and do not necessarily represent those of their affiliated organizations, or those of the publisher, the editors and the reviewers. Any product that may be evaluated in this article, or claim that may be made by its manufacturer, is not guaranteed or endorsed by the publisher.

## Supplementary Material

Supplementary Figure 1 Pseudotime trajectories of hepatic CD8^+^ T cells from TG mice. **(A, B)** Differentiation trajectory generated using Monocle2 with all hepatic CD8^+^ T cells. Cells are colored according to three states **(A)**, and the plot is split by cell subpopulations, which partition the trajectory **(B)**. **(C)** Correlation analysis of differential states of effector memory CD8^+^ T subpopulations.

Supplementary Figure 2 Metabolic characteristics of CD8αα and proliferation effector memory CD8^+^ T cells from TG mice. **(A, B)** GSEA analysis of glucose metabolism pathway **(A)** and lipid metabolism pathways **(B)** between CD8αα and proliferation effector memory CD8^+^ T cells from TG mice. The normalized enrichment score (NES), original p value and FDR adjusted p values (q.value) are shown. **(C)** Scaled average expression of glycolysis, ATP synthesis and oxidative phosphorylation molecules for CD8^+^ T subpopulation of CD8αα and proliferation effector memory CD8^+^ T cells from TG mice.

Supplementary Figure 3 CD8αα effector memory T cells, a subpopulation with more tissue resident memory like. **(A)** Vlnplot shows the genes downregulated *(Klf2, Tcf7, S1pr1)* and upregulated *(Cxcr6, Itga1, Cxcr3)* in tissue-resident memory T cells from TG mice. **(B)** Scaled average expression of the functional genes and transcription factors of tissue-resident memory T cells for each CD8^+^ memory T subpopulations from TG mice. Individual cells are represented on the horizontal axis and grouped by cluster. **(C)** Normalized score of signatures upregulated in human tissue-resident memory T cells of effector memory CD8^+^ T cells from TG mice, including *ITGA1, CRTAM, PDCD1, DUSP6, IFNG, IL2, IL17A, IL10, CXCR6, CD101, CD69* and *ITGAE*.

Supplementary Figure 4 Compared with CD8αβ T cells, CD8αα T cells had a more avtivated phenotype. **(A)** Representative FACS plot showing the composition of hepatic CD8αβ and CD8αα T cells in TG mice. **(B, C)** Frequency of CD8αβ and CD8αα T cells in TG mice that express IFNγ and TIGIT.
