# Supplementary figures and images for: Single-Cell Characterization of Hepatic CD8+ T Cells in a Murine Model of Primary Biliary Cholangitis
Source: Front Immunol. 2022 Apr 20;13:860311. doi: 10.3389/fimmu.2022.860311 (PMC9065443; doi:10.3389/fimmu.2022.860311)

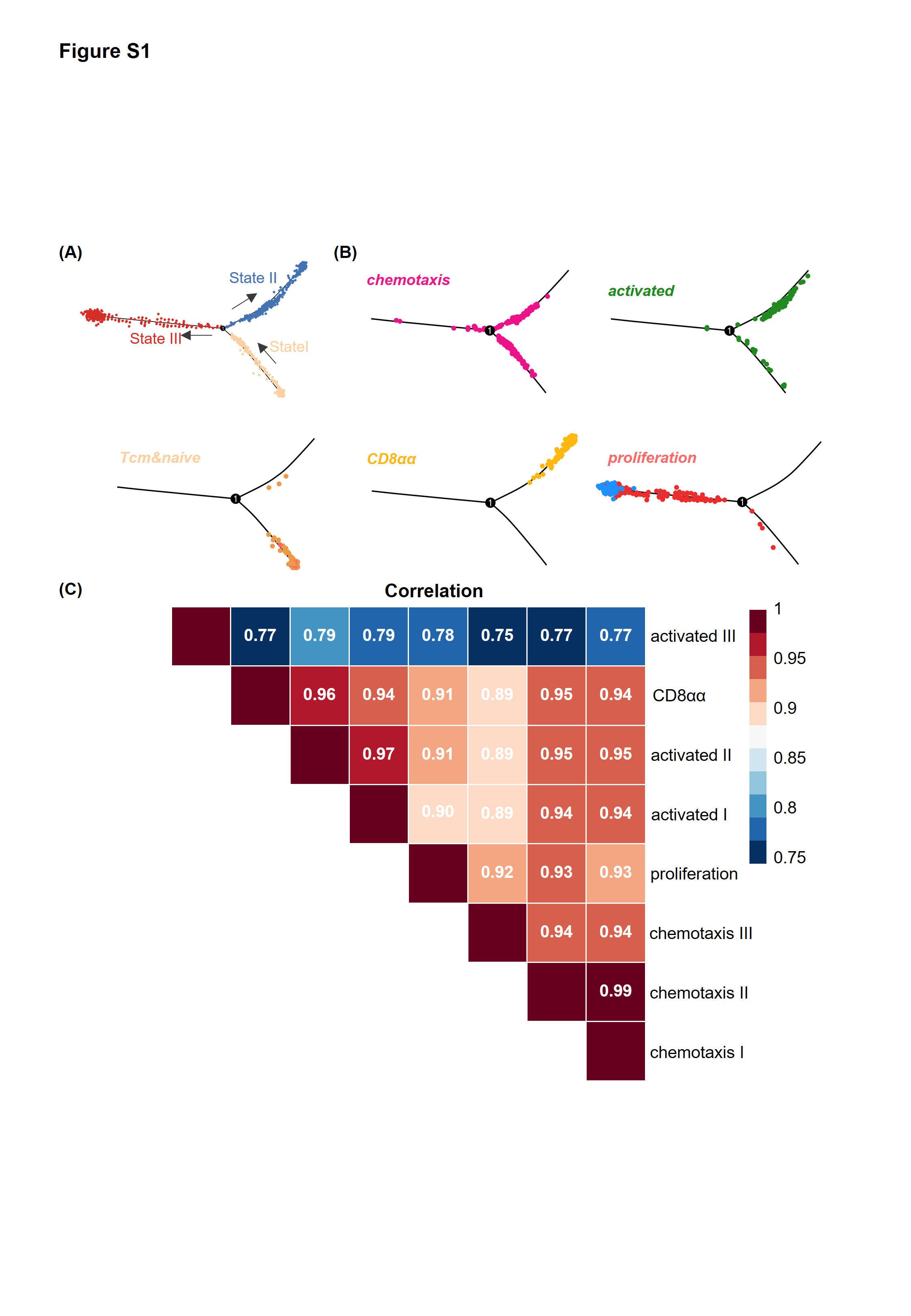

Supplement: Supplementary Figure 1 — Pseudotime trajectories of hepatic CD8+ T cells from TG mice. (A, B) Differentiation trajectory generated using Monocle2 with all hepatic CD8+ T cells. Cells are colored according to three states (A), and the plot is split by cell subpopulations, which partition the trajectory (B). (C) Correlation analysis of differential states of effector memory CD8+ T subpopulations. [file Image_1.tif]

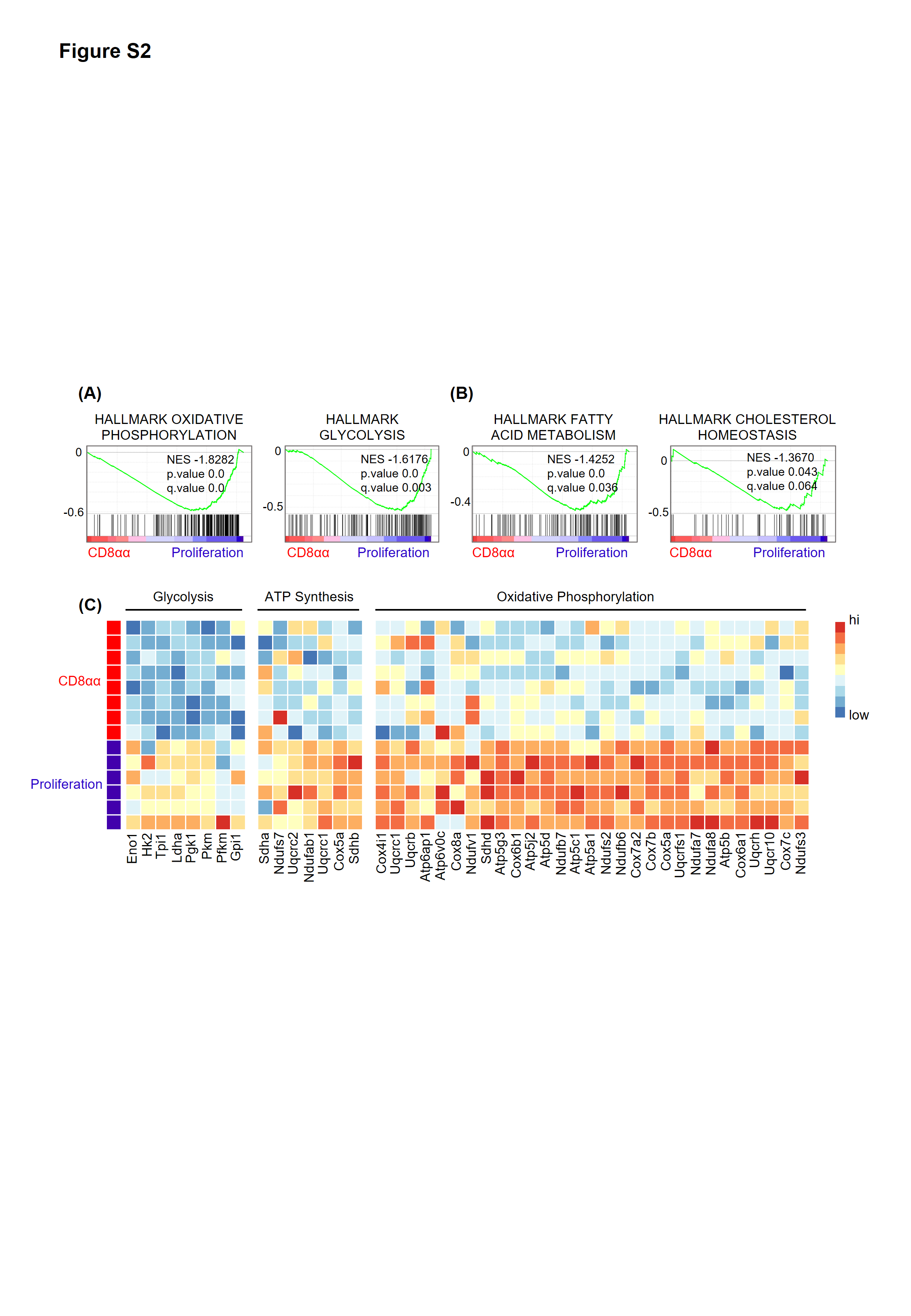

Supplement: Supplementary Figure 2 — Metabolic characteristics of CD8αα and proliferation effector memory CD8+ T cells from TG mice. (A, B) GSEA analysis of glucose metabolism pathway (A) and lipid metabolism pathways (B) between CD8αα and proliferation effector memory CD8+ T cells from TG mice. The normalized enrichment score (NES), original p value and FDR adjusted p values (q.value) are shown. (C) Scaled average expression of glycolysis, ATP synthesis and oxidative phosphorylation molecules for CD8+ T subpopulation of CD8αα and proliferation effector memory CD8+ T cells from TG mice. [file Image_2.tif]

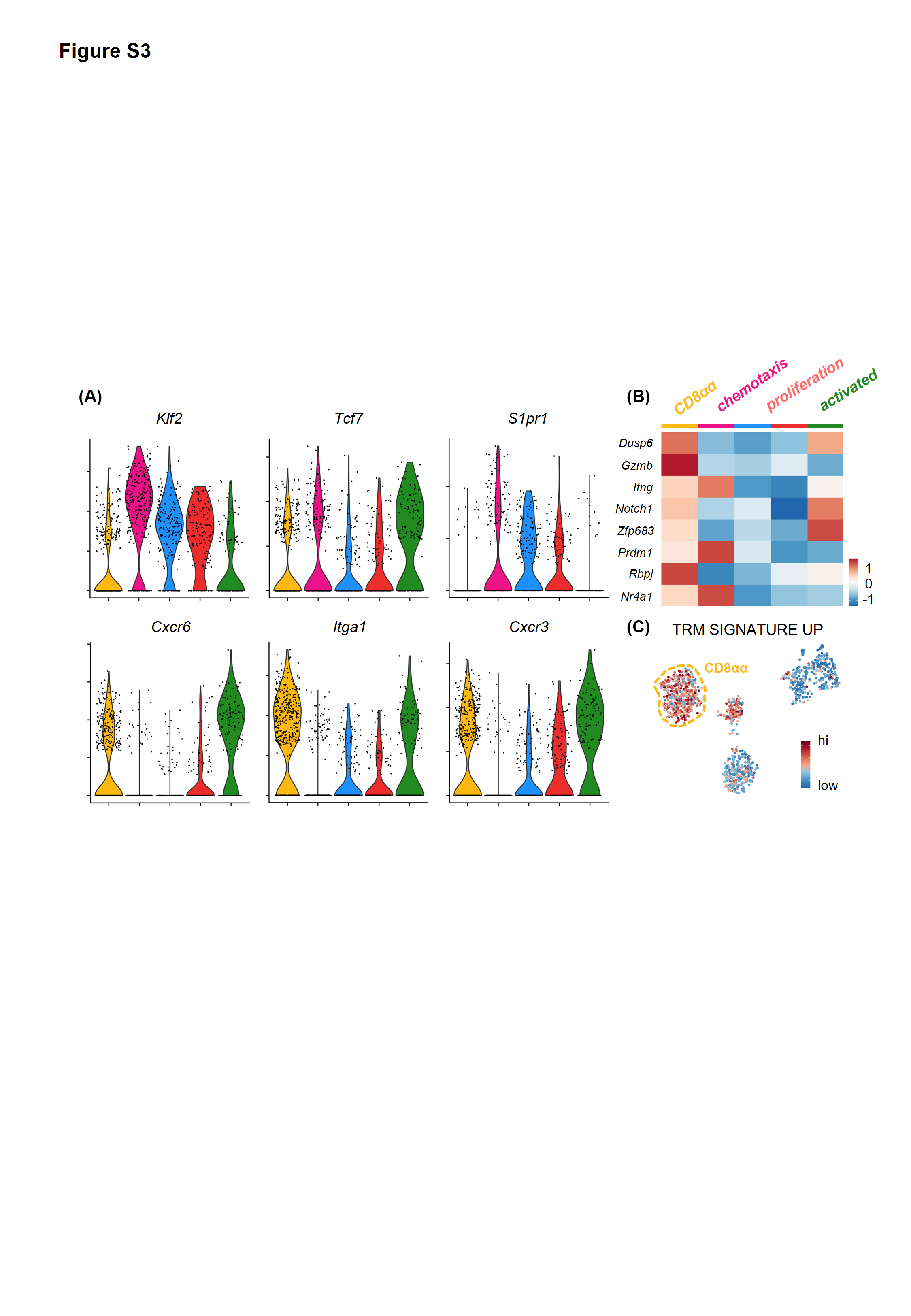

Supplement: Supplementary Figure 3 — CD8αα effector memory T cells, a subpopulation with more tissue resident memory like. (A) Vlnplot shows the genes downregulated (Klf2, Tcf7, S1pr1) and upregulated (Cxcr6, Itga1, Cxcr3) in tissue-resident memory T cells from TG mice. (B) Scaled average expression of the functional genes and transcription factors of tissue-resident memory T cells for each CD8+ memory T subpopulations from TG mice. Individual cells are represented on the horizontal axis and grouped by cluster. (C) Normalized score of signatures upregulated in human tissue-resident memory T cells of effector memory CD8+ T cells from TG mice, including ITGA1, CRTAM, PDCD1, DUSP6, IFNG, IL2, IL17A, IL10, CXCR6, CD101, CD69 and ITGAE. [file Image_3.tif]

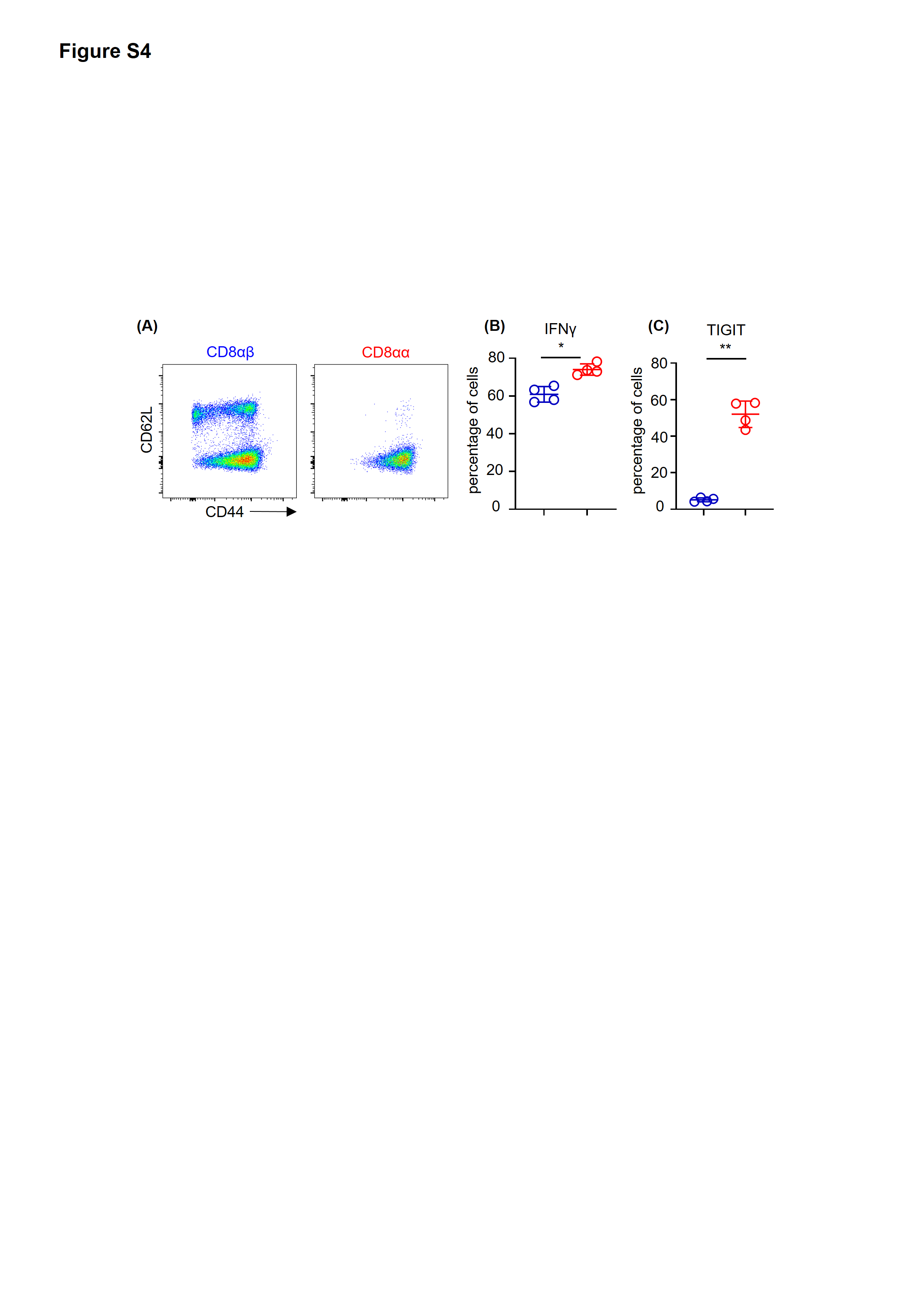

Supplement: Supplementary Figure 4 — Compared with CD8αβ T cells, CD8αα T cells had a more avtivated phenotype. (A) Representative FACS plot showing the composition of hepatic CD8αβ and CD8αα T cells in TG mice. (B, C) Frequency of CD8αβ and CD8αα T cells in TG mice that express IFNγ and TIGIT. [file Image_4.tif]
